# Application and removal of polyanionic microbicide compounds enhances subsequent infection by HIV-1

**DOI:** 10.1186/1743-422X-9-33

**Published:** 2012-01-26

**Authors:** Vanessa Pirrone, Shendra Passic, Brian Wigdahl, Fred C Krebs

**Affiliations:** 1 Department of Microbiology and Immunology, and Center for Molecular Therapeutics and Resistance, Center for Sexually Transmitted Disease, Institute for Molecular Medicine and Infectious Disease, Drexel University College of Medicine, Philadelphia, PA 19102, USA

**Keywords:** AIDS, HIV-1, Microbicide, Polyanion, Carrageenan, Cellulose sulfate, Enhancement

## Abstract

**Background:**

Continued efforts are being directed toward the development of microbicides that will be used to reduce or eliminate the risk of HIV-1 sexual transmission. Unfortunately, clinical trials involving polyanion-containing microbicide formulations, including Carraguard (λ-carrageenan [LC]) and Ushercell (cellulose sulfate [CS]) demonstrated that these products were ineffective and may have, in some circumstances, increased the risk of HIV-1 infection. These findings prompted reassessments of the in vitro activities of these agents to determine whether variables that can affect agent safety and efficacy had been overlooked during preclinical testing. One such variable is product retention and loss following topical application.

**Results:**

In the present studies involving an HIV-1-susceptible cell line and primary human immune cells, product loss was mimicked by introducing and then removing polyanionic compounds prior to HIV-1 infection. In these in vitro "washout" experiments, LC and CS significantly enhanced HIV-1 infection, despite potent antiviral activity when introduced simultaneously with the virus. The presence and magnitude of this effect were dependent on compound identity and concentration; target cell; interval between compound removal and virus challenge; and coreceptor usage. Levels of enhancement (relative to controls) were considerable, exceeding a 200% increase (CS) in P4-R5 MAGI cells and a 300% increase (LC) in human peripheral blood mononuclear cells.

**Conclusions:**

These studies, which demonstrate significant increases in HIV-1 infection subsequent to application and removal of LC and CS, support plausible explanations for the failures of microbicides formulated from these compounds. Detailed studies are now underway to determine the mechanism responsible for this enhancement effect and to assess the potential contribution of this effect to the clinical failures of these agents.

## Background

In the present AIDS pandemic, approximately 50% of the 33.4 million people infected with HIV-1 are women [1]. This fact highlights the urgent need for female-controlled and female-centric prevention methods that can effectively reduce or eliminate the risk of infection in women. One response to this need is the development of safe and effective topical microbicides, which are chemical entities that, when applied prior to vaginal- or rectal-receptive intercourse, will prevent the transmission of HIV-1 [2-6]. Numerous candidate microbicide compounds with varied and diverse mechanisms of action are under preclinical development. In addition, numerous microbicides have been or are being evaluated in clinical trials for safety and efficacy.

Among the potential microbicide agents advanced through clinical trials were two compounds classified as polyanions: carrageenan and cellulose sulfate (CS). λ-Carrageenan (LC) was thought to be especially promising as an antiviral agent because its demonstrated in vitro IC_50 _(the concentration at which 50% virus inhibition is achieved) was more than 100-fold lower than its anticoagulant threshold, suggesting a margin of safety for use at concentrations capable of inhibiting infection [7]. Indeed, initial phase I and II clinical trials indicated that a mixture of κ- and λ-carrageenan (Carraguard) was safe for vaginal application [8-12]. However, in phase III trials, with time to seroconversion as the measure of efficacy, Carraguard was shown to be ineffective as an anti-HIV-1 microbicide [13].

CS, which was formulated into a microbicide known as Ushercell, was shown in vitro to have broad activity against HIV-1, herpes simplex virus types 1 and 2, *Neisseria gonorrhoeae*, and *Chlamydia trachomatis *[14]. After numerous clinical trials that established the safety of Ushercell [15-20], two separate phase III trials were initiated to assess its efficacy against HIV-1 transmission. Both trials were halted when, in one trial, an increased prevalence of HIV-1 infection was detected among women using Ushercell [21]. Although subsequent analyses indicated that the apparent increase in HIV-1 infection was not statistically significant, neither trial suggested any effectiveness of Ushercell as an inhibitor of HIV-1 transmission [21,22].

As a result of the clinical failures of Carraguard and Ushercell, efforts have been intensified to identify and understand factors that may adversely affect the in vivo efficacy of microbicides containing polyanionic compounds. Such studies have revealed that seminal plasma can decrease the antiviral activity of the polyanionic compounds PRO2000 and CS [23] and that exposure to CS or nonoxynol-9 (N-9) can disrupt an epithelial barrier and increase HIV-1 passage through the epithelial barrier as well as increase viral replication [24]. In studies designed to reevaluate the antiviral activity of CS, enhancement of HIV-1 infection by subefficacious concentrations of CS was proposed as a contributor to the failure of Ushercell [25]. Although the validity of the observed enhancement was challenged [26,27], other groups have independently observed similar effects of exposure to polyanionic agents, including enhancement of TZM-bl cell infection by low concentrations of Carraguard [28]; augmentation of dendritic cell-mediated infection by Carraguard [28]; and "modest" increases in replication at low concentrations of PRO2000 and the anionic, dendrimer-based SPL7013. However, the effects noted in this latter study did not appear to be limited to polyanionic molecules, since a similar trend was also observed after application of the entry inhibitor enfuvirtide [29].

Enhanced HIV-1 infection in the presence of low concentrations of a compound [25,28] suggests another variable that may adversely affect microbicide efficacy during clinical trials: product loss following topical application. As a result of microbicide loss after application, concentrations of the active agent in the female reproductive tract will diminish over time [30]. Indeed, product leakage (directly measured or reported by study participants) was noted in phase I and phase II trials of carrageenan and CS [15,31-34]. Once the concentrations of the active agents have dropped sufficiently to render them ineffective as microbicides, mechanisms that result in enhanced HIV-1 infection at low or negligible concentrations (such as those described above) may prevail.

To investigate this hypothesis, we infected cells in vitro with HIV-1 after exposure to and removal of LC, CS, or dextran sulfate (DS). DS was included as a prototypical polyanionic compound with established activity against HIV-1 [35-39]. This experimental design, which has been used to investigate persistent or "memory" antiviral activity [35,40-44], was used in these studies to mimic the loss of these agents after topical vaginal application. In experiments involving an HIV-1-susceptible indicator cell line and primary human immune cells, HIV-1 infection was significantly enhanced by prior exposure to these compounds. Furthermore, the timing and degree of enhancement were dependent on target cell, co-receptor phenotype, compound identity and concentration, and the timing of the viral challenge. These results suggest a role for the host cell in polyanion-dependent enhancement of HIV-1 infection.

## Results

### Polyanionic compounds effectively inhibit infection by HIV-1 BaL and IIIB

**Figure 1:**
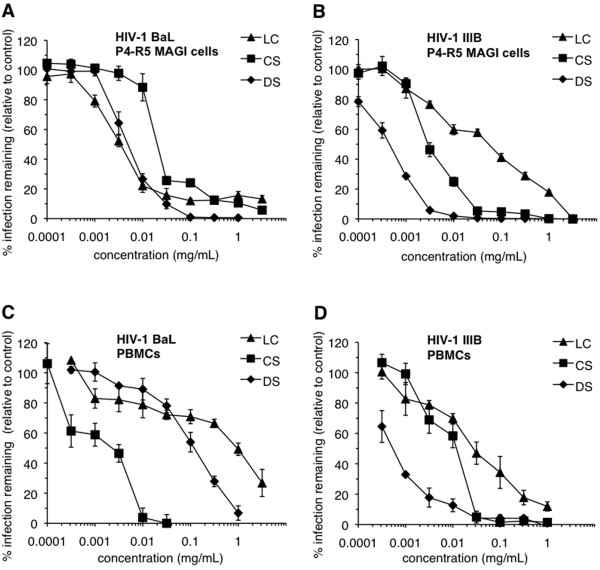
**Polyanionic compound antiviral activity is demonstrated against both R5 and X4 HIV-1 in both P4-R5 MAGI cells and peripheral blood mononuclear cells (PBMCs)**. P4-R5 MAGI cells were infected for 1 h with (**A**) HIV-1 strain BaL (CCR5 tropic) or (**B**) HIV-1 strain IIIB (CXCR4 tropic) in the presence of dextran sulfate (DS), cellulose sulfate (CS), or λ-carrageenan (LC). Infectivity remaining is expressed relative to mock-treated, HIV-1-infected cells. PBMCs were incubated for 1 h with (**C**) HIV-1 strain BaL or (**D**) IIIB in the presence of DS, CS, or LC. Infectivity remaining is expressed relative to mock-treated, HIV-1-infected cells. Data for this experiment are shown as mean values and calculated standard deviations from two independent assays in which each concentration was examined in triplicate. Error bars represent standard deviations.

To establish compound concentrations to be used in the washout assays, P4-R5 MAGI cells and peripheral blood mononuclear cells (PBMCs) were exposed to each compound and simultaneously infected with HIV-1. Concurrent introduction of LC, CS, or DS resulted in concentration-dependent inhibition of infection of the P4-R5 MAGI cell line by the R5 HIV-1 strain BaL (Figure 1A) or the X4 strain IIIB (Figure 1B). Similarly, concentration-dependent antiviral activity was observed in experiments involving infection of primary human PBMC populations with HIV-1 BaL (Figure 1C) or IIIB (Figure 1D). These results were used to calculate IC_50 _and IC_90 _concentrations for each compound (Table 1). Despite similarities in compound charge and mechanisms of antiviral activity [45], antiviral activities (as indicated by IC_50 _and IC_90_) appeared to vary with target cell, compound, and virus co-receptor usage (Table 1).

**Table 1:** Calculated IC_50 _and IC_90 _for λ-carrageenan, cellulose sulfate, and dextran sulfate (mg/mL)

	P4-R5 MAGI				Human PBMCs			
	
	HIV-1 BaL		HIV-1 IIIB		HIV-1 BaL		HIV-1 IIIB	
	
	IC_50_	IC_90_	IC_50_	IC_90_	IC_50_	IC_90_	IC_50_	IC_90_
LC	0.0037 (± 0.0009)	6.04**^a ^**(± 0.79)	0.064 (± 0.0084)	1.96 (± 0.077)	0.967 (± 0.53)	3.16**^b ^**(± 0.04)	0.061 (± 0.021)	0.87 (± 0.32)

CS	0.023 (± 0.0018)	1.24 (± 0.47)	0.003 (± 0.00047)	0.026 (± 0.00073)	0.0026 (± 0.0003)	0.0091 (± 0.0052)	0.014 (± 0.0034)	0.03 (± 0.0025)

DS	0.0058 (± 0.0011)	0.031 (± 0.012)	0.00052 (± 0.00011)	0.0028 (± 0.00015)	0.14 (± 0.045)	0.9 (± 0.18)	0.00056 (± 0.00017)	0.014 (± 0.0064)

*CS *cellulose sulfate, *DS *dextran sulfate, *IC**_50 _***the concentration at which 50% virus inhibition is achieved, *IC**_90 _***the concentration at which 90% virus inhibition is achieved, *LC *λ-carrageenan, *PBMCs *peripheral blood mononuclear cells

^a^Extrapolated concentration

^b^LC concentrations > 3.16 mg/mL could not be used due to excessive solution viscosity. Because 90% inhibition of HIV-1 BaL infection by LC in PBMCs was not achieved, the LC IC_75 _(concentration that resulted in a 75% reduction of infection with respect to mock-treated, HIV-1-infected cells) was calculated and used in the PBMC washout experiments

Presented data are from two independent experiments in which each data point was examined in triplicate

### Preexposure to CS or LC enhances P4-R5 MAGI infection by R5 HIV-1

**Figure 2:**
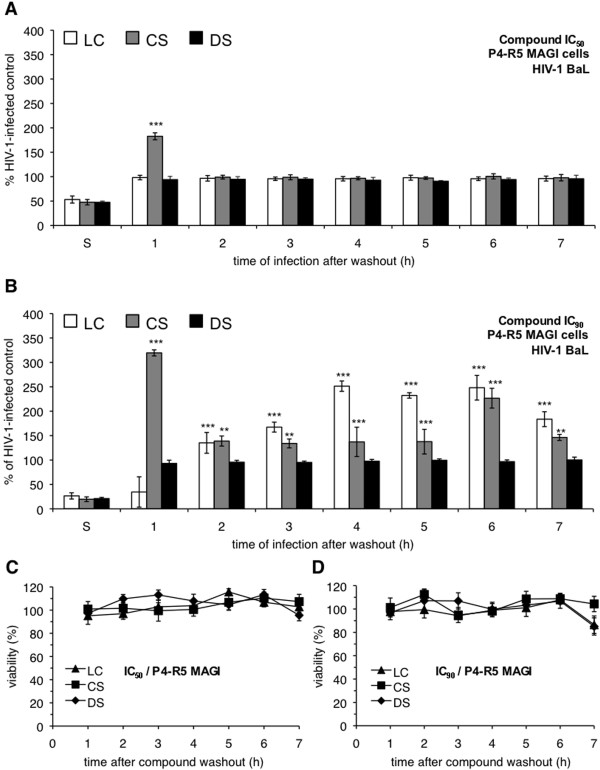
**Preexposure of P4-R5 MAGI cells to select polyanionic compounds results in enhancement of R5 HIV-1 infection**. P4-R5 MAGI cells were incubated for 1 h with (**A**) IC_50 _or (**B**) IC_90 _(the concentrations at which 50% or 90% virus inhibition is achieved, respectively) dextran sulfate (DS), cellulose sulfate (CS), or λ-carrageenan (LC). Following exposure, cells were washed thoroughly and either challenged immediately with HIV-1 strain BaL for 1 h or supplemented with new medium for up to 6 h before infection with HIV-1 BaL (as described in Materials and Methods). Infectivity remaining is expressed relative to mock-treated, HIV-1-infected cells. To assess possible changes in cell viability associated with compound washout, P4-R5 MAGI cells were incubated for 1 h with (**C**) IC_50 _and (**D**) IC_90 _concentrations of DS, CS, or LC. The IC_50 _and IC_90 _for BaL inhibition were used because these concentrations were, with one exception, higher than the IIIB IC_50 _and IC_90 _(Table 1). Cells were subsequently assessed for viability as described in Materials and Methods. Viability is expressed relative to mock-treated cells. Data for this experiment are shown as mean values and calculated standard deviations from two independent assays in which each concentration was examined in triplicate. Error bars represent standard deviations. **p ≤ 0.01, *** *p *≤ 0.001.

In the washout infection experiments, antiviral compounds were removed from the culture media before introduction of virus. As a consequence, inhibitors that act on viral binding and entry, like the polyanionic compounds in question [46-48], were expected to be ineffective in this assay, because they would not be present at the time of infection. Indeed, DS, when introduced at either its IC_50 _(Figure 2A) or IC_90 _(Figure 2B), was effective only when present during the addition of virus ("S"). DS had no effect on HIV-1 infection if it was added and then subsequently removed from the cells before introduction of virus.

The activities of LC and CS in these experiments, however, were unexpected. At its IC_50_, CS washout resulted in a 60% increase in infectivity above the control level of HIV-1 infection when it was removed from cells 1 h before infection by HIV-1 BaL but not when its removal preceded infection by 2-7 h (Figure 2A). The magnitude of enhancement appeared to be concentration-dependent, because washout of CS at its IC_90 _subsequently caused a greater than 200% increase in infection at 1 h after washout (Figure 2B). Furthermore, the effect of the higher concentration of CS was time-dependent: The higher concentration of CS caused modest increases in infection (~40% increase) at 2, 3, 4, 5, and 7 h after washout and a larger increase (~140% increase) at 6 h after washout.

Enhancement of HIV-1 infection by LC was also concentration- and time-dependent. Like DS, the removal of LC at its IC_50 _had no subsequent effect on HIV-1 BaL infection (Figure 2A). However, washout of LC at its IC_90 _caused significant increases in HIV-1 BaL infection that were first observed 2 h after washout (Figure 2B). The enhancement appeared to reach a plateau 4-6 h after compound removal (~140% increase) and was somewhat less (~80% increase) at 7 h. Interestingly, HIV-1 infection was still inhibited by LC at 1 h after exposure.

To verify that the observed enhancement of infection was not associated with changes in cell viability during the course of the washout experiments, P4-R5 MAGI cell viability was assessed after washout of each compound at its HIV-1 BaL IC_50 _and IC_90 _(because these concentrations were, with one exception, higher than the IIIB IC_50 _and IC_90_). No changes in P4-R5 MAGI cell viability were observed out to 7 h after washout (Figure 2C, D). These results indicated that cell viability was not altered by compound washout and was not a confounding factor in these experiments.

### Preexposure to CS or LC causes limited increases in P4-R5 MAGI infection by X4 HIV-1

**Figure 3:**
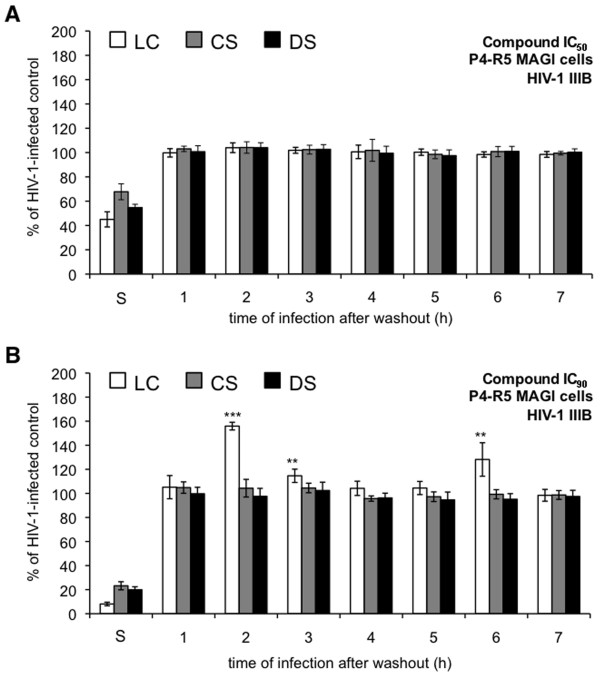
**Exposure to λ-carrageenan (LC) results in enhanced infection of P4-R5 MAGI cells by X4 HIV-1**. P4-R5 MAGI cells were incubated for 1 h with (**A**) IC_50 _or (**B**) IC_90 _(the concentrations at which 50% or 90% virus inhibition is achieved, respectively) of dextran sulfate (DS), cellulose sulfate (CS), or LC. Following exposure, cells were washed thoroughly and either challenged immediately with HIV-1 strain IIIB for 1 h or supplemented with new medium for up to 6 h before challenge with HIV-1 IIIB (as described in Materials and Methods). Infectivity remaining is expressed relative to mock-treated, HIV-1 infected cells. Data for this experiment are shown as mean values and calculated standard deviations from two independent assays in which each concentration was examined in triplicate. Error bars represent standard deviations. **p ≤ 0.01; *** *p *≤ 0.001.

To investigate the possibility that enhanced infection subsequent to polyanionic compound exposure was dependent on HIV-1 co-receptor usage, similar experiments were performed using HIV-1 IIIB (Figure 3). None of the compounds at their respective IC_50 _had any effect on infections initiated after compound washout (Figure 3A). In experiments involving each compound at its IC_90 _(Figure 3B), LC was the only compound that had an effect after washout (~60% increase at 2 h and ~30% increase at 6 h). A comparison of the effects of compound exposure and washout on infection by HIV-1 BaL and IIIB suggests that co-receptor usage can affect enhancement of HIV-1 infection by polyanionic compounds.

### Infection of primary human immune cells by HIV-1 BaL is increased by preexposure and removal of polyanionic compounds

**Figure 4:**
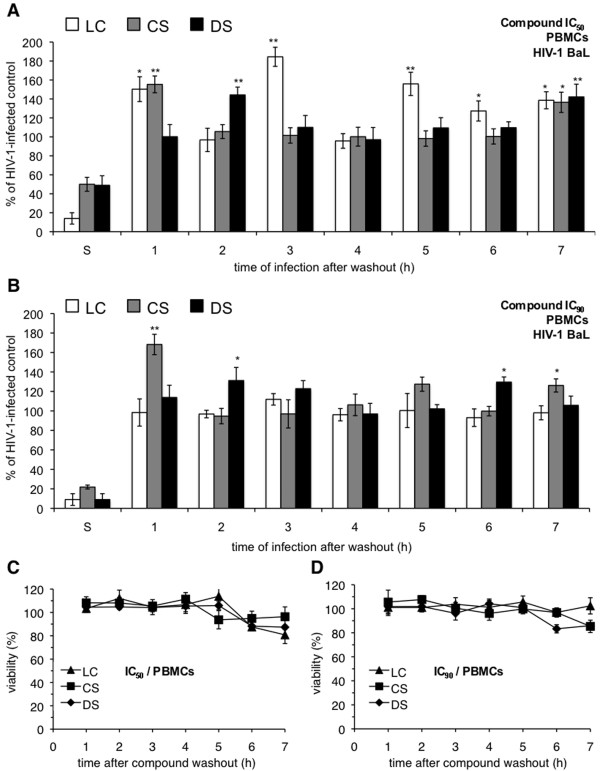
**Enhanced infection by HIV-1 BaL is observed in peripheral blood mononuclear cells (PBMCs) following exposure to select polyanionic compounds**. PBMCs were incubated for 1 h with (**A**) IC_50 _or (**B**) IC_90 _(the concentrations at which 50% or 90% virus inhibition is achieved, respectively) dextran sulfate (DS), cellulose sulfate (CS), or λ-carrageenan (LC). Following exposure, cells were washed thoroughly and either challenged immediately with HIV-1 strain BaL for 1 h or supplemented with new medium for up to 6 h before challenge with HIV-1 BaL (as described in Materials and Methods). Infectivity remaining is expressed relative to mock-treated, HIV-1 infected cells. To assess possible changes in cell viability associated with compound washout, PBMCs were incubated for 1 h with (**C**) IC_50 _and (**D**) IC_90 _concentrations of DS, CS, or LC. The IC_50 _and IC_90 _for BaL inhibition were used because these concentrations were, with two exceptions, higher than the IIIB IC_50 _and IC_90 _(Table 1). Cells were subsequently assessed for viability as described in Materials and Methods. Viability is expressed relative to mock-treated cells. Data for this experiment are shown as mean values and calculated standard deviations from two independent assays in which each concentration was examined in triplicate. Error bars represent standard deviations. *p ≤ 0.05; **p ≤ 0.01.

Experiments similar to those described above were performed to verify that the enhancement effects of polyanionic compounds could be observed during HIV-1 infection of primary human immune cell populations and were not limited to a cell line susceptible to HIV-1 infection. In washout experiments involving HIV-1 BaL infection of PBMCs, all three compounds caused varying degrees of enhancement (Figure 4). In contrast to experiments involving P4-R5 MAGI cells, DS washout resulted in modest enhancements of HIV-1 infection, with ~40% increases in infection at 2 and 7 h after washout at its IC_50 _(Figure 4A) and ~30% increases at 2 and 6 h after washout at its IC_90 _(Figure 4B). CS washout caused similar increases in HIV-1 infection at 1 and 7 h after washout at both concentrations, ranging from ~30% to ~70% over controls. Washout of LC at its IC_50 _caused increases in infection up to ~80% at 1, 3, 5, and 7 h after washout. However, LC at its IC_75 _(used in lieu of an IC_90_, which could not be determined; see Table 1) had no effect on HIV-1 BaL infection. As was demonstrated using the P4-R5 MAGI cell line, compounds applied to and removed from PBMCs had no subsequent effect on cell viability (Figure 4C, D).

**Figure 5:**
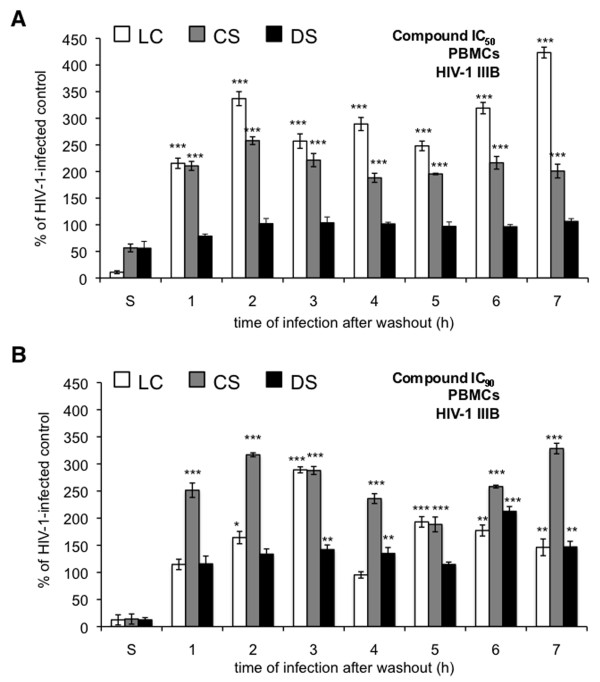
**Enhanced infection by HIV-1 IIIB is observed in peripheral blood mononuclear cells (PBMCs) following exposure to select polyanionic compounds**. PBMCs were incubated for 1 h with (**A**) IC_90 _or (**B**) IC_50 _(the concentrations at which 50% or 90% virus inhibition is achieved, respectively) dextran sulfate (DS), cellulose sulfate (CS), or λ-carrageenan (LC). Following exposure, cells were washed thoroughly and either infected immediately with HIV-1 strain IIIB for 1 h or supplemented with new medium for up to 6 h before infection with HIV-1 IIIB (as described in Materials and Methods). Infectivity remaining is expressed relative to mock-treated, HIV-1-infected cells. Data for this experiment are shown as mean values and calculated standard deviations from two independent assays in which each concentration was examined in triplicate. Error bars represent standard deviations. *p ≤ 0.05; **p ≤ 0.01; ***p ≤ 0.001.

Unlike experiments involving HIV-1 IIIB infection of P4-R5 MAGI cells, washout experiments using PBMCs and HIV-1 IIIB revealed more substantial increases in HIV-1 infection (Figure 5). Washout of all three compounds caused increases in HIV-1 infection relative to mock-exposed control infections. Although DS had no effect on HIV-1 IIIB infection at its IC_50 _(Figure 5A), washout of DS at its IC_90 _(Figure 5B) caused significant increases in infection at 3, 4, 6, and 7 h after washout (up to 113% increase at 6 h). Washout of the CS IC_50 _caused increases at all times after washout (up to 323% increase at 7 h). Similar increases were observed after washout of the CS IC_90 _(up to 228% increase at 7 h). Enhancement following LC washout at both concentrations was also observed. However, increases after washout of LC at its IC_50 _were generally higher, with maximal enhancement at 2 h (237% increase) and 7 h (323% increase).

## Discussion

Investigations into the failures of N-9, C31G, Ushercell, Carraguard, and PRO2000 continue to provide valuable insights into factors that may adversely impact in vivo microbicide efficacy. Studies of N-9, which began shortly after the phase III trial failures [49], focused on cervicovaginal tissue damage and inflammation following application of N-9. These studies implicated cervical tissue damage [50], induced recruitment of immune cell populations [50], and the production of proinflammatory cytokines, such as interleukin (IL)-1β, IL-6, and IL-8 [51-53] as explanations for the general failure of an N-9-based microbicide, as well as increases in HIV-1 infection after high-frequency application [54]. In contrast, factors that can be invoked to explain the failures of the remaining microbicides have not yet been clearly identified. It is apparent, however, that multiple mechanisms are likely associated with the failures of these microbicides.

The present in vitro studies, which were conducted to examine microbicide loss following topical application as a potential mechanism of microbicide failure, demonstrated significant increases in HIV-1 infection following the application and removal of the polyanionic compounds DS, CS, and LC. Increases in infection, which were observed in experiments using an HIV-1-susceptible cell line as well as primary human immune cells, were found to be dependent on the target cell and co-receptor usage by the infecting virus.

Although enhancement of HIV-1 infection following polyanionic compound washout was clearly demonstrated in the HIV-1 indicator cell line and in primary human immune cells, specific results were not always comparable between experiments using these two cell populations. For example, LC washout at its IC_50 _had no effect on HIV-1 IIIB infection of P4-R5 MAGI cells (Figure 3A) but had a significant effect on HIV-1 IIIB infection of PBMCs (Figure 5A). Conversely, LC application and removal at its IC_90 _(Figure 2A) resulted in enhancement of HIV-1 BaL infection that increased with post-exposure time in P4-R5 MAGI cells, whereas the comparable experiment in PBMCs (Figure 4A) demonstrated no HIV-1 BaL enhancement as a consequence of LC pre-exposure. These apparently disparate results are likely due to differences inherent in the cells used in these experiments, including cell type (recombinant cervical carcinoma cell line versus primary human immune cell) and levels of receptor and co-receptor expression (over-expressed on the P4-R5 MAGI cells). In addition, a considerably lower multiplicity of infection (MOI) was used in the PBMC experiments. Any or all of these factors could have affected the outcome of these experiments. Future mechanism-focused experiments may provide more definitive insights into the underlying causes of these differences in outcome.

The effects of compound washout were also dependent on the interval between compound removal and introduction of virus, suggesting both immediate and delayed mechanisms of enhanced infection. Enhancement following a short (1- to 2-h) interval between compound removal and infection suggests immediate changes on the cell surface that enhance the early stages of the HIV-1 replication cycle. Cell surface changes initiated by microbicide exposure may be induced by the retention of residual amounts of compound at the cell surface or may persist after the compound has been removed. Interactions between compound and cell surface may enhance HIV-1 infection by favoring increased viral binding and entry. Although numerous cell surface molecules have been shown to participate in HIV-1 binding and entry, the impact of co-receptor usage on enhancement implicates the involvement of the HIV-1 co-receptors CCR5 and CXCR4. However, the specificity of this effect remains to be demonstrated.

Alternatively, enhancement after a relatively long (6- to 7-h) interval after washout may suggest more indirect mechanisms potentially involving signal transduction events initiated by compound exposure. As evidence that polyanionic molecules can initiate cell signaling cascades, studies of innate immune activation using both human and murine model systems have demonstrated that carrageenan-induced inflammation requires signaling through Toll-like receptor 4 [55,56] as well as MyD88-dependent signal transduction [57]. Polyanion-induced signal transduction and inflammation do not appear to be limited to carrageenan, because DS has been used for 20 years as an agent that reliably induces experimental colitis in mice [58]. The demonstrated activities of these polyanionic molecules raise the possibility that delayed increases in HIV-1 infection observed in the present studies were the result of signaling initiated by DS, CS, or LC. Because of the time required to initiate events downstream of compound-initiated signal transduction, the effects of this mechanism of enhancement were not immediately evident but did affect HIV-1 infection after a period of delay. A specific target of polyanion-mediated signaling within the viral replication cycle was not apparent from these results and will need to be determined as part of future investigations.

In the experiments involving primary human PBMCs cells, observations of post-exposure, time-dependent enhancement may also have been complicated by cell population heterogeneity. Differences in the levels and mechanisms of enhancement between T lymphocytes and cells of monocyte lineage, which also differ greatly in frequency within the total cell population, may have biased the observed time-dependent enhancement of HIV-1 infection relative to results obtained using the homogeneous P4-R5 MAGI cell line. For example, washout of LC at its BaL IC_50 _(Figure 4A) resulted in enhanced infection at 1, 3, 5, 6, and 7 h post-exposure and no enhancement at 2 and 4 h. This unusual pattern of enhancement might be explained as an overlay of time-dependent enhancement in T cells and monocytes/macrophages, with different peaks of infection arising from different HIV-1-infected cell populations. Furthermore, if compound-mediated intracellular signaling results in the downstream release of cytokines and chemokines, the combined pattern of time-dependent enhancement might be altered further because the release of these soluble factors subsequently affects the susceptibility to infection of uninfected neighboring cells. The contributions of these factors will need to be resolved in experiments using enriched target cell populations.

The present results, as well as the results of previously published studies, indicate that enhancement under these conditions is also dependent on the identity of the compound. This same assay design was previously used to evaluate the antiviral activity of the biguanide-based cationic entry inhibitor NB325 [35] as well as the activities of the polyanionic compounds poly (styrene-*alt*-maleic acid) (*alt*-PSMA), poly(styrene sulfonate) (PSS), and DS [59]. In direct contrast to the present studies, which were focused on DS, CS, and LC, washout experiments involving NB325 demonstrated persistent protection from infection by HIV-1 IIIB after compound removal. Furthermore, studies involving *alt*-PSMA and PSS demonstrated that enhancement of infection following compound washout is not an effect that can be generally ascribed to polyanionic compounds. Although *alt*-PSMA and PSS are similarly based on polystyrene backbones, they derive their polyanionic charges from different moieties: maleic acid in the case of *alt*-PSMA and sulfonic acid in PSS. In washout assays, *alt*-PSMA removal had no effect (enhancement or inhibition) on HIV-1 infection, whereas PSS significantly increased levels of infection relative to infection controls. The results of our previous studies with DS are consistent with previously published studies [29,60,61], which demonstrated that DS could enhance HIV-1 infection in monocyte-derived macrophages but not in CD4+ T lymphocytes [35]. The current experiments demonstrated DS-mediated enhancement in PBMCs but not in P4-CCR5 MAGI cells, presumably due to the inclusion of monocyte-derived macrophages in the former cell population.

How does one reconcile the present results with the clinical failures of these compounds? Low concentrations of compound will be achieved clinically by dilution during sexual intercourse or time-dependent compound leakage after application. Early phase I and phase II safety and acceptability trials of carrageenan and CS indicated, either through direct measurement or through self-reporting by the women in the trials, that the product leaked after application [15,31,34,62,63]. Leakage would decrease the concentration of active compounds available in the cervicovaginal environment, negating the antiviral activity of the microbicide and potentially favoring mechanisms that promote HIV-1 infection. Therefore, the efficacy of a polyanionic compound such as carrageenan or CS would be dictated by concentration. At high concentrations (as found after the initial application of the microbicide product), the antiviral activity of the compound will override any enhancing activity. At low concentrations (or after the complete loss of microbicide), the compound would be unable to inhibit infection or offset the mechanisms that enhance infection, and its overall effect would be to increase the risk of infection. The activity of such a compound in a clinical trial would be driven by variables that affect the amount of product in the cervicovaginal tract, including the elapsed time between product application and sexual intercourse, dilution by seminal fluid, and the degree of product leakage following application.

These results also suggest that plans to use carrageenan as a potential "active excipient" in formulations of other active agents may not be advisable. Although the safety of Carraguard was demonstrated in early clinical trials and no enhancement was evident in the results of the failed phase III trial [8-10,12,13,64], the possibility remains that, under different circumstances, the use of carrageenan in a formulated microbicide may result in an increased risk of HIV-1 transmission. In light of these results, the use of carrageenan in future microbicide products should be carefully considered. These results, however, do not broadly rule out the use of polyanionic molecules in antiviral formulations, because enhancement of HIV-1 infection does not appear to be a universal property of polyanionic compounds. Previous studies of the polyanionic *alt*-PSMA demonstrated that its washout did not result in increased infection [37].

## Conclusions

These studies, which demonstrate significant increases in HIV-1 infection subsequent to application and removal of λ-carrageenan and CS, support plausible explanations for the failures of microbicides formulated from these compounds (and, perhaps, also PRO2000). These investigations also emphasize the need for careful, continued scrutiny of candidate microbicide compounds for activities that may counter their efficacy against HIV-1 infection and transmission.

## Materials and methods

### Compounds

DS (Dextralip 50, catalog # D8787, lot 71K1378) and λ-carrageenan (catalog #C-3889, lot 122K1444) were purchased from Sigma-Aldrich (St. Louis, MO). CS (catalog #AC17781-0500, lot A0219391) was purchased from ACROS Organics (Morris Plains, NJ).

### Cell line maintenance and primary cell isolation

P4-R5 MAGI cells (NIH AIDS Research and Reference Reagent Program #3580) were maintained in Dulbecco modified Eagle medium supplemented with 10% fetal bovine serum, sodium bicarbonate (0.05%), antibiotics (penicillin, streptomycin, and kanamycin at 40 μg/mL each), and puromycin (1 μg/mL) [65]. Human PBMCs were isolated from whole blood (Biological Specialty Corp., Colmar, PA) using Ficoll-hypaque (Amersham Biosciences, Piscataway, NJ) density gradient centrifugation and were subsequently cultured in RPMI (Roswell Park Memorial Institute) medium supplemented with 10% fetal bovine serum, sodium bicarbonate (0.05%), antibiotics (penicillin and streptomycin at 90 μg/mL each), phytohemagglutinin-P (Sigma-Aldrich, catalog #L8754 5 μg/mL), and IL-2 (NIH AIDS Research and Reference Reagent Program #11697; 20 U/mL) [66]. After 48 h, PBMCs were washed and incubated for an additional 24 h prior to infection in the absence of phytohemagglutinin-P.

### Assessing polyanionic compound inhibition of cell-free HIV-1 infection

P4-R5 MAGI cells were cultured 18 h prior to infection at a density of 1.2 × 10^4 ^cells/well in a 96-well plate. Cells were incubated for 1 h with HIV-1 BaL (1.0 MOI) or IIIB (0.1 MOI) (Advanced Biotechnologies, Inc., Columbia, MD) in the absence or presence of LC, CS, or DS. After 1 h, cells were washed, cultured for an additional 46 h, and subsequently assayed for HIV-1 infection using the Galacto-*Star *β-galactosidase reporter gene assay system for mammalian cells (Applied Biosystems, Bedford, MA).

Human PBMCs, stimulated as described above, were seeded at a density of 1 × 10^5 ^cells/well in a 96-well plate. Cells were then incubated for 1 h with HIV-1 BaL (0.6 MOI) or IIIB (0.3 MOI) in the absence or presence of LC, CS, or DS. After 1 h, cells were washed and subsequently cultured for 3 d, at which time the cells were washed and supplied with new medium supplemented with IL-2. The cells were then incubated for an additional 3 d and subsequently assayed for HIV-1 production by determining the level of p24 core antigen in the supernatant using an HIV-1 p24 antigen enzyme-linked immunosorbent assay (ELISA) (ZeptoMetrix, Buffalo, NY). Levels of infection were expressed relative to mock-treated, HIV-1-infected cells.

### Evaluating polyanionic compound anti-HIV-1 activity in a washout assay

P4-R5 MAGI cells were incubated with each compound for 1 h at its IC_50 _or IC_90 _(compound concentrations that caused 50% or 90% reductions, respectively, in infection relative to mock-treated, HIV-1-infected cells). Cells were then challenged with HIV-1 BaL (1.0 MOI) or IIIB (0.1 MOI) for 1 h at 37°C either concurrently with compound incubation or up to 7 h after compound removal ("washout" by three consecutive cell washes) and incubation in new medium without compound. Following infection, cells were washed, incubated for approximately 48 h, and then assayed for HIV-1 production as described above. Stimulated PBMCs were assessed for enhanced HIV-1 infection in a washout assay similar to the P4-R5 MAGI cell-based assay with HIV-1 BaL (0.6 MOI) and IIIB (0.3 MOI). Following compound washout and subsequent infection, cells were washed, incubated for 6 d, and then assayed for HIV-1 production using a p24 enzyme-linked immunosorbent assay as described above.

### Evaluating the effect of compound washout on cell viability

P4-R5 MAGI cells were seeded at a density of 4 × 10^4 ^cells/well in a 96-well plate approximately 18 h prior to experiment. Cells were then exposed to the indicated concentrations of LC, CS, or DS for 1 h. Following the exposure period, cells were washed and assessed for viability using a 3-(4,5-dimethylthiazol-2-yl)-2,5-diphenyltetrazolium bromide assay [67,68] of viability at the indicated times after washout of the antiviral compounds. In assays involving PBMCs, cells were seeded at a density of 1 × 10^5 ^cells/well in a 96-well plate and subsequently exposed to LC, CS, or DS for 1 h. Following the exposure period, cells were washed and assessed for viability using a 3-(4,5-dimethylthiazol-2-yl)-5-(3-carboxymethoxyphenyl)-2-(4-sulfophenyl)-2H-tetrazolium assay [69,70] of viability at the indicated times after washout. In both assays, viability following compound exposure was determined relative to mock-exposed cells.

### Data analyses

Data for all experiments are shown as mean values and calculated standard deviations from two independent assays in which each concentration was examined in triplicate. IC_50 _and IC_90 _calculations were performed using the Forecast function of Microsoft Excel. Statistical significance was calculated in comparison with mock-exposed cells (unless otherwise indicated) using a two-tailed, unpaired Student *t *test.

## Abbreviations

CS: Cellulose sulfate; DS: Dextran sulfate; IL: Interleukin; LC: λ-carrageenan; MOI: Multiplicity of infection; N-9: Nonoxynol-9; PBMC: Peripheral blood mononuclear cell; *alt*-PSMA: Poly(styrene-*alt*-maleic acid); PSS: Poly(styrene sulfonate).

## Competing interests

The authors declare that they have no competing interests.

## Authors' contributions

VP completed viral and toxicity assays of the peripheral blood mononuclear cells, completed all data and statistical analyses, and drafted the manuscript. SP completed all of the P4-R5 MAGI cell viral and toxicity assays. BW helped conceive the study, participated in its design and coordination, and helped draft the manuscript. FCK conceived the study, participated in its design and coordination, and helped draft the manuscript. All authors read and approved the final manuscript.
